# Supplementary material for: A sensorimotor model shows why a spectral jamming avoidance response does not help bats deal with jamming
Source: eLife. 2020 Jul 28;9:e55539. doi: 10.7554/eLife.55539 (PMC7406351; doi:10.7554/eLife.55539)
Supplement: Source data 1. [file elife-55539-data1.pdf]

BatModel– Readme

1. Extract the winrar folder to your computer.

2. Open MATLAB®:

3. Set the path on MATLAB command window to ‘**BatModel**’ (the folder thee code files were extracted to):

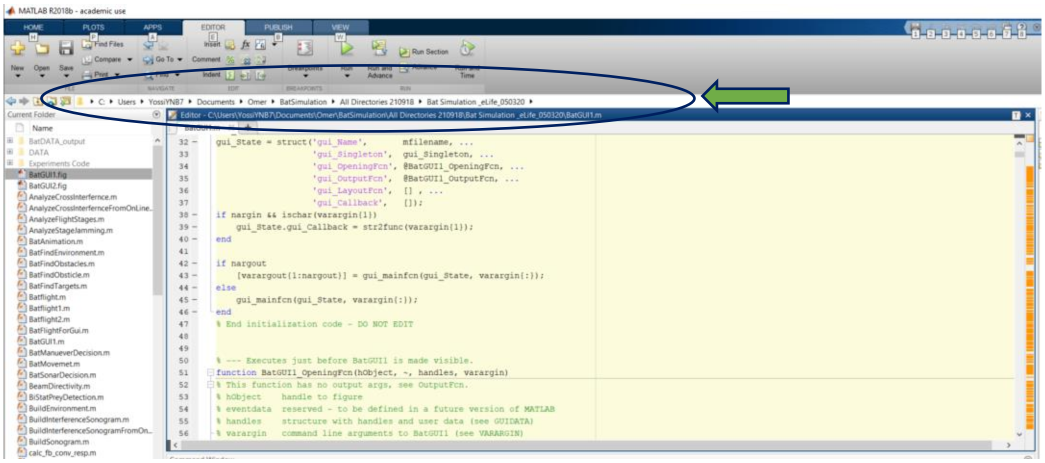

4. Type **BatGUI1** on the Command window.
   This will open the Graphical User Interface that controls and executes the simulation:

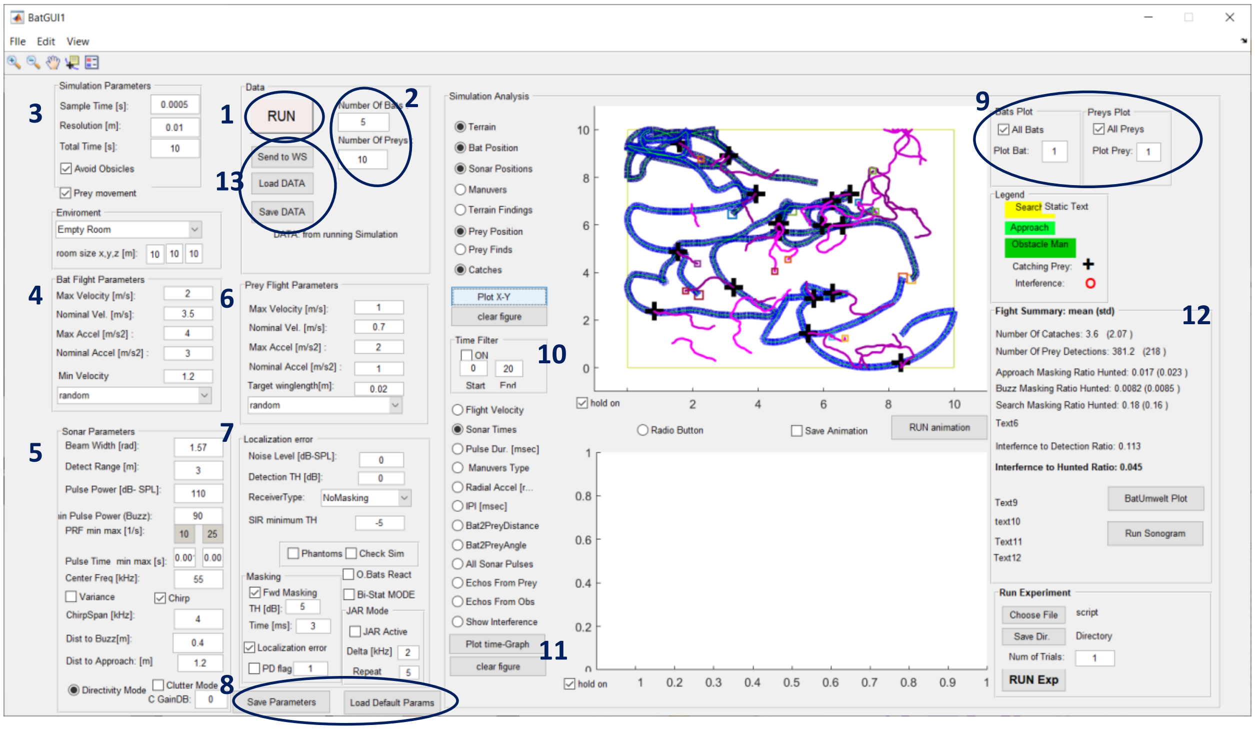

5. Run the Simulation:
   5.1. **(2)** Set how many bats and prey items participate the simulation.
   5.2. **(3-7)** Change the parameters of the simulation.

5.3. **(8)** Load or save the parameters of the simulation.

Important: Saving the parameters will change the default parameters.

5.4. **(1)** Press '**Run**' to execute the simulation.

6. Analyze the data:
   6.1. **(9)** After executing the simulation, decide which bats and prey items to plot.
   6.2. **(10)** Decide which parameter to plot in the x-y figure, and press 'Plot X-Y'.
   6.3. **(11)** Decide which parameters to plot in the time graph, and press 'Plot time graph'. Use the hold on check button.
   6.4. **(12)** Main statistics of the trial: mean values and standard deviations (in brackets).

7. Further Analysis:
   7.1. **(13)** The output data of the simulation is stored at structure called '**BatDATA**'. Press 'send to WS' to send the output to the MATLAB work-space.It is possible to load and save the output data as files.
   7.2. BatDATA consists the following fields:

```
BatDATA = struct with fields:
        PREY: [1×(number_of_prey_items) struct]
        BAT: [1×( number_of_bats) struct]
        AllParams: [1×1 struct]
        FlightInterferceSummary: [1×1 struct]
        FilterBank: [1×1 struct]
```

   7.3. Main data to analyze:
      7.3.1. **BatDATA.AllParams** – the parameters used in the simulation run (i.e. a flight)
      7.3.2. **BatDATA.FlightInterferceSummary** – main statistics regrading masking and jamming.
      7.3.3. **BatDATA. BAT(x). InterReportStrctOnLine** – detailed data for each bat.

   7.4. Main Functions and files:
      7.4.1. **BatGUI1.m** – runs the Graphical User Interfaces
      7.4.2. **BatFlightForGui.m** – The main function that executes the simulation and calls other function to caclulate all the required data.
      7.4.3. **…\DATA\ DefaultParameters.mat** – a MATLAB file with the required parameters
      7.4.4. **… BatDATA_output\ BatData_04-Mar-2020_224159.mat** – an example of an output struct after running the simulation
      7.4.5. **…\Experiments Code*.m** - some examples of code for running the model numerous times with different parameters.

Good Luck,

Omer Mazar,

Emai: Omer_mazar@yahoo.com

March 2020
